# Giant Cell Tumor of the Wrist After Fracture Osteosynthesis: A Case Report

**DOI:** 10.7759/cureus.34110

**Published:** 2023-01-23

**Authors:** Francisco Rodriguez Fontan, Diana Douleh, Andrew Federer, Bennie Lindeque

**Affiliations:** 1 Department of Orthopedics, University of Colorado Anschutz Medical Campus, Aurora, USA

**Keywords:** cryoablation, tissue biopsy, bone lesion, distal radius fracture open reduction internal fixations, giant-cell tumor of bone

## Abstract

A 60-year-old female sustained a distal radius fracture and underwent open reduction internal fixation with a volar locking plate. The patient had an uneventful recovery until four months postoperatively when the patient clinically regressed, and an expansile, radiolucent metaepiphyseal lesion was found. Further workup revealed this was a giant cell tumor of bone (GCTB). Definitive management consisted of extensive curettage, cryoablation, and cementation of the lesion, and the hardware was left intact. The current case presents an uncommon presentation of GCTB. The case illuminates the importance of thorough scrutiny of postoperative radiographs when clinical improvement plateaus or regresses and the need to pursue additional workup when the clinical course is atypical. The authors query the possibility of a sub-radiological presentation of GCTB.

## Introduction

Giant cell tumor of bone (GCTB) is an osteolytic, often benign, but locally aggressive lesion, accounting for approximately 4-6% of primary bone tumors and 20% of benign skeletal tumors [1]. It is often encountered as an incidental or fracture-related eccentric lytic metaepiphyseal lesion around the knee and distal radius [2]. Distal radius fractures are commonly encountered by the orthopaedist (one-sixth of all fractures) and oftentimes require open reduction internal fixation (ORIF) when indicated [3,4]. In the setting of a GCTB, this can present as a pathological fracture, and treatment options, as well as prognosis regarding recurrence rate, vary substantially [5].

Only two prior cases in the literature presented the development of distal radius GCTB lesions after patients undergoing ORIF for distal radius fractures with the apparent absence of inciting pathological lesions at the time of index injury [6,7].

This article briefly reviews the epidemiology, diagnosis, treatment options, and recurrence rate for GCTB. More notably, in the setting of a case report of a GCTB lesion presenting at four months postoperatively from a distal radius fracture ORIF in a 60-year-old female patient. ﻿The patient was informed that data concerning the case would be submitted for publication, and she provided consent.

## Case presentation

**Figure 1:**
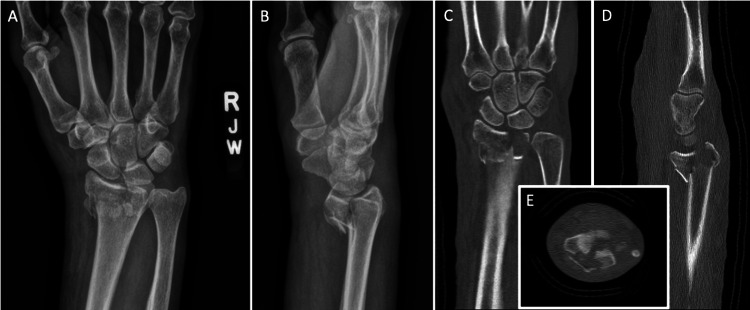
Injury films and preoperative CT scan A-B) anteroposterior and lateral X-rays views of the right wrist: comminuted volar Barton distal radius fracture with loss of radial height and volar radial tilt. C-E) coronal, sagittal, and axial views of CT scan showing comminuted fracture pattern, depressed articular surface.

**Figure 2:**
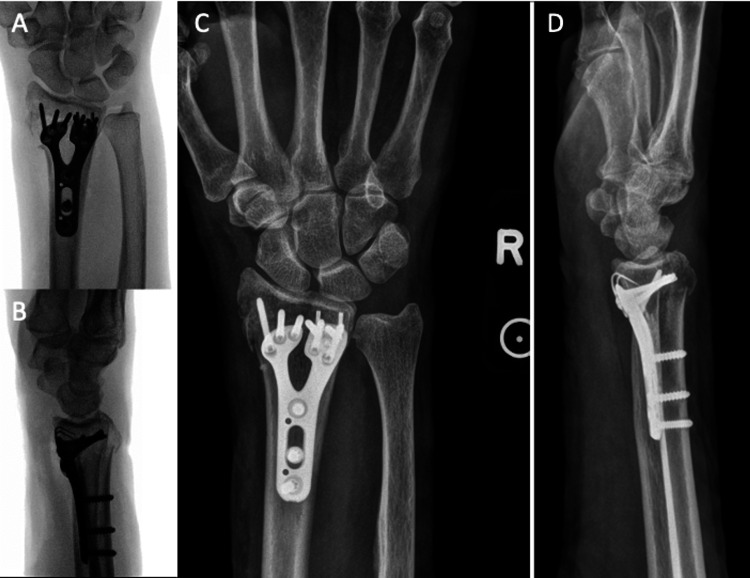
Intraoperative and postoperative films. A-B) intraoperative fluoroscopy. C-D) two-week postoperative anteroposterior and lateral X-rays views of the right wrist: volar plate and hook plate appearing intact, with the restoration of the articular surface, radial height, and radial tilt.

A 60-year-old female presented to an outpatient orthopaedic clinic in September 2021 with a one-week history of acute, traumatic right wrist pain after a mechanical ground-level fall while walking her dog. A distal radius fracture had been identified at an urgent care facility on the date of injury at which time the patient was provisionally splinted. The patient is an otherwise healthy, right-hand dominant female, non-smoker with noncontributory past medical/surgical history. The patient reported no prior history of trauma to the right wrist. On a focused musculoskeletal exam, she presented with a closed injury associated with significant swelling, but no visible deformity over the dorsum of the wrist. She was neurovascularly intact. Radiographs demonstrated a right, comminuted distal radius volar Barton fracture (Figure 1). At that time, a joint decision between the orthopaedist and the patient was to proceed with ORIF. Given significant comminution, a pre-operative computed tomography (CT) scan was obtained for surgical planning (Figure 1). The surgery took place two weeks following the date of injury. It was performed with a standard volar approach to the wrist and fixation was achieved with a 3.5 mm volar locking plate, and a hook plate (Skeletal Dynamics, Miami, FL) (Figure 2). Postoperatively, the patient initially progressed without complication, achieving a nearly symmetric range of motion (ROM) to the contralateral wrist at 12 weeks while working with a hand therapist. 

**Figure 3:**
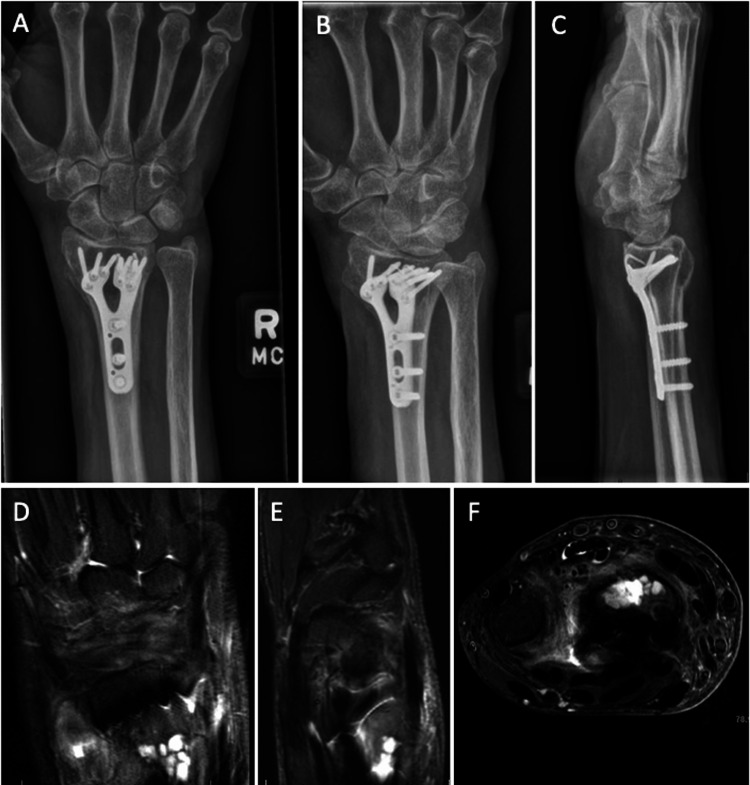
Follow-up X-rays and MRI A-C) Four-month postoperative anteroposterior, oblique, and lateral X-ray views of the right wrist: metaphyseal lucency, intact hardware, and some loss of radial height. D-F) coronal, sagittal, and axial T2 MRI sequence showing dorsal metaphyseal hyperintense lesion.

At a four-month routine clinical follow-up, in mid-January 2022, the patient presented with regression of ROM, as well as pain and swelling. She was afebrile with stable vital signs and was healthy-appearing. She expressed tenderness over the first and second extensor compartments and more wrist stiffness, concerning for intersection syndrome. Radiographs demonstrated no evidence of plate or screw prominence dorsally that could cause inflammation or tenosynovitis. A radial metaphyseal lucency was found on radiographs (Figure 3). At that time, bone resorption, infection, or cyst were considered in the differential diagnoses. Inflammatory markers, including CRP and ESR, were normal. A magnetic resonance imaging (MRI) was done and showed no signs of tenosynovitis or vascular aneurysm. Proximal to Lister’s tubercle, a periosteal reaction and a peripheral enhancing cyst were found on the T2 sequence (Figure 3), the latter measuring approximately 7 x 8 x 8 mm. With normal inflammatory markers, a diagnosis of infection was considered less likely.

Over the next two months, the patient reported worsening dorsal wrist tenderness with some additional pain at the base of the thumb, mostly with pinching activities. Additional workup included an ultrasound (US) to better evaluate the flexor and extensor tendons, given the concern for tendon irritation due to the plate. This showed no signs of tenosynovitis. As for the cyst, the US was concerning for a possible giant cell tumor and revealed progression of the expansive lesion with cortical thinning.

Upon interdisciplinary team discussion with Orthopaedic Oncology, the decision was made to proceed with a biopsy for pathology and culture. A biopsy was performed through a small open dorsal approach, radial to Lister’s tubercle. When incising the retinaculum overlying the second extensor compartment a non-purulent, brown fluid was encountered. The dorsal cortical aspect of the metaphyseal radius was found thinned and insignificant. Sharp excisional debridement was performed in the metaphysis through the dorsal cortical window. Several samples, including soft tissue, bone, and fluid were sent for pathology and culture. The hardware was not revised and was found to be stable.

**Figure 4:**
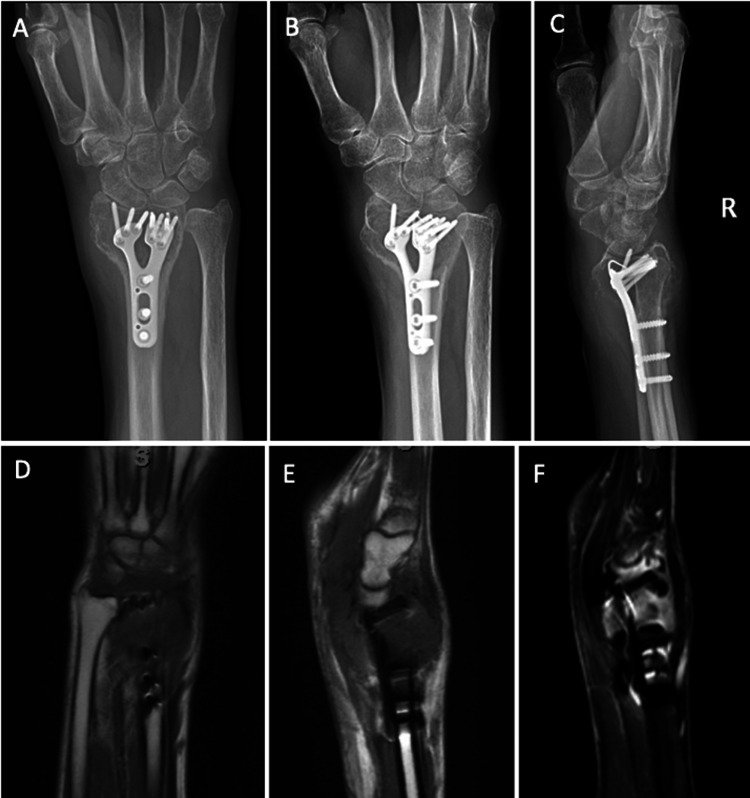
Follow-up X-rays and MRI A-C) Five-month postoperative anteroposterior, oblique, and lateral X-rays views of the right wrist: enlarged metaphyseal lucency with a dorsal cortical appearing breach, intact hardware, and continued loss of radial height. D-F) T1 coronal, T1 sagittal, and FS T2 sagittal MRI sequences showing enlarged, dorsal metaphyseal lesion T1 hypointense and T2 hyperintense classic for giant cell.

She was seen thereafter by the Orthopaedic Oncology team. On exam, she had an unremarkable healing incision with swelling over the dorsal and volar aspect of the wrist. Shortly after the biopsy, the pathology report was conclusive for GCTB. Cultures remained negative throughout the postoperative course. A chest X-ray was ordered which was indicative of no lung metastasis. A new MRI was obtained for re-evaluation prior to definitive surgery and showed a T1 hypointense and T2 hyperintense expansile lesion with cortical destruction, measuring 3 x 2.4 x 4.2 cm (Figure 4). No other bone lesions were found around the radius and ulna.

**Figure 5:**
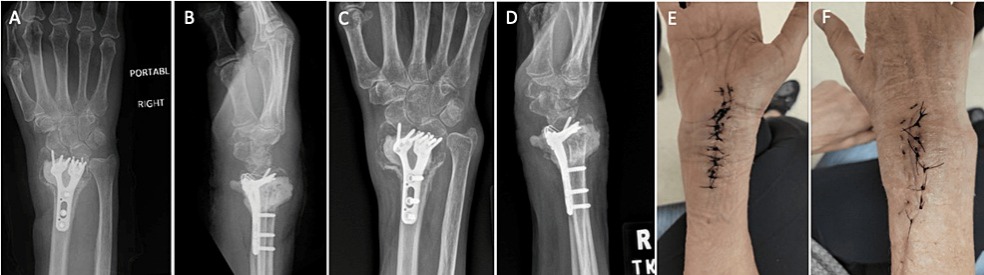
Postoperative X-rays and follow-ups. A-B) immediate postoperative anteroposterior and lateral X-rays; C-D) four-week postoperative anteroposterior and lateral X-ray views of the right wrist: hardware and cement appearing intact. There is residual loss of radial height radial, tilt is neutral, with unchanged bone resorption and lucency at the cement bone interface. Cement extends dorsally and volarly due to cortical breach. E-F) Volar and dorsal right wrist incisions with sutures in place at three weeks postoperatively.

One week after the biopsy, she underwent definitive surgery. A dual volar and dorsal approach was performed with extensive tenolysis (flexor tendons), carpal tunnel release, neurolysis (median and ulnar nerve), and extensive curettage of the radial metaphyseal tumor. Soft tissue and deep bone biopsy were sent for cultures and pathology. These were again positive for GCTB (CD 68+) with no signs of malignancy. Cytogenetic testing showed no chromosomal aberrations. While protecting soft tissue structures, stepwise cryoablation (volar cavity two cycles for seven minutes, and dorsal capsule one cycle for five minutes) and cementation with tobramycin of the cavity followed. The hardware was found stable and in a good position, and thus the decision was made to leave it in place. Postoperatively at four weeks, May 2022, she had an uneventful recovery. She was found neurovascularly intact throughout. The dorsal incision was well-healed, and the volar incision had no signs of dehiscence or infection but showed delayed healing (all sutures were kept for three to four weeks), and minimal swelling (Figure 5). At that time, ROM was still limited but improving with therapy. She was maintained on non-weight bearing (NWB) precautions with a splint for comfort and nighttime use.

## Discussion

Most GCTB occur in the 20-40-year-old age range with a slight female predominance as described previously [1]. The third-most common location is the distal radius (10%). The histological hallmark is the osteoclast-like multinucleated giant cells with associated mononuclear stromal cells [8]. Despite its benign nature, it is locally aggressive and variably recurrent, and on some occasions presents with lung metastasis (1-4%) [2]. Hence, it’s often challenging to manage and requires routine surveillance for recurrence. 

Radiologically, the common presentation is an eccentric, lytic lesion extending into the subchondral bone. Other features are cortical thinning, expansile remodeling, periosteal reaction, extension from the metaphysis, fluid levels, or cystic spaces [1,9]. Based on radiological findings, the differential diagnosis, among others, should include primarily osteomyelitis, chondroblastoma, aneurysmal bone cyst, multiple myeloma, and bone metastasis (e.g., thyroid, renal) [1,9].

The goals of management are eradication with limb preservation, maintenance of function, and prevention of recurrence. Historically, curettage alone has been reported to have a recurrence rate of 21-65%, yet this has been substantially, but variably, reduced with co-adjuvant therapies (polymethylmethacrylate <29%, cryoablation 0-20%, phenol 3-33%, high-speed burr, iodine), which often combine options (e.g., cryoablation or phenol and PMMA) [9]. Some researchers advocate that due to the higher recurrence rate in the distal radius [10], en-bloc resection with reconstruction would be, theoretically, a better option given its lower recurrence rate (<12%) [2,11]. However, this may come with a significant tradeoff for function, ROM, and higher complication rate [10]. Zou et al. in a recent retrospective study on high-grade GCTB distal radius comparing extensive curettage and high-speed burring versus wide resection and reconstruction found no difference in recurrence rate regarding surgical technique (27% vs. 23.8%) [10]. Independent risk factors for local relapse were the size of the tumor ≥ 5 cm and soft tissue extension. Moreover, the curettage group achieved better functional outcomes [10]. In terms of surveillance of recurrence, in the case of curettage and cementing, it is important to trace the cement bone interface and look for new or progressing lucencies [1].

For the purpose of this case report, we found two unusual prior unique cases that presented as distal radius GCTB after ORIF [6,7]. Marshal et al. presented a case of a 77-year-old female who underwent ORIF for a distal radius fracture. This patient underwent hardware removal for presumed tendon irritation 1.5 years later. Of note, both the treating orthopaedist and radiologists commented on this patient's marked osteopenia intraoperatively and radiologically; however, a radiologically well-defined lesion was not observed. At the time of hardware removal, an expansile lesion was encountered and the intraoperative pathology report was confirmatory for GCTB. At that time the lesion was curetted, and cement was provisionally placed. A second surgery went on to en-bloc resection with osteoarticular allograft reconstruction [7]. A more recent case, outlined by Siesel et al., reported on a 32-year-old pregnant female who developed a GCTB six years after sustaining a distal radius fracture and undergoing ORIF. In this setting, she had new onset of pain after a fall but no hardware failure. The lesion appeared to be dorsal and associated with soft tissue mass. An intraoperative frozen section during hardware removal confirmed the diagnosis, and definitive treatment consisted of curettage, high-speed burring, cement filling, and new plate fixation [6]. Both mentioned cases had an unremarkable two-year recovery with no recurrences. Our case appeared shortly after initial fixation, and in retrospect, a subtle radiological suggestion for possible dorsal lesions could be made prior to the initial surgery, but both the orthopaedist and radiologist did not find any pathological lesion leading to the fracture. Intraoperatively, no pathological lesion or atypical tissue was found at the time of initial fixation. The same consideration, in retrospect, was arisen by the authors in the case of the 77-year-old woman case in which, the surgeon noted severe osteopenic bone intraoperatively and the radiologist reported marked osteopenia on CT, but no definitive delineated lesion [7]. In our case, curettage, and cement were the treatment of choice, and tobramycin was added to the cement to mitigate the risk of infection in the treatment for GCTBs after extensive surgery (2-25%) [1]. At one month postoperatively, this patient demonstrated intact hardware and cement-bone interface with no concern for recurrence at that time. 

## Conclusions

To conclude, given the scarce literature in case reports regarding GCTB after fracture, it raises the concern or question if there is a sub-radiological presentation for a pathological fracture. In other words, whether a possible precipitating lesion would be structurally sufficient to weaken the bone in a way that would not initially be captured radiologically on plain X-rays and or on CT scans, which upon injury and fixation would trigger a progressive and expansile lesion. On the contrary, there is no known association between GCTB arising after a fracture. 
